# Comparing the effects of traditional resistance training and functional training on the bio-motor capacities of female elite taekwondo athletes

**DOI:** 10.1186/s13102-023-00754-9

**Published:** 2023-10-20

**Authors:** Leila Khazaei, Abdolhossein Parnow, Sadegh Amani-shalamzari

**Affiliations:** 1 Sport-Biosciences Department, Physical Education and Sport Sciences Faculty, Razi University, University Street, Kermanshah, 6414414874 Iran; 2 Exercise Physiology Department, Faculty of Sport Sciences, Kharazmi University, Tehran, Iran

**Keywords:** Aerobic power, Muscle power, Martial Art, Strength, Balance

## Abstract

**Background:**

The capabilities of the biomotors are essential to the success of the taekwondo athlete. This study aimed to compare eight weeks of functional training and traditional resistance on the bio-motor capacities of elite female taekwondo athletes.

**Methodology:**

Seventeen elite taekwondo athletes (mean age = 21.7 years, mean height = 167.2 cm and mean weight = 60.8 kg) volunteered to participate in the study. They were randomly divided into two groups: functional training (FT) and traditional resistance training (TRT). Participants trained for 8 weeks, 3 sessions per week, for 75–90 min. Aerobic power, anaerobic power, speed, reaction time, agility, muscle power, dynamic balance, flexibility, upper and lower body muscle strength, core and upper body muscle endurance, and blood lactate level were measured before and after training programs.

**Results:**

Non-significant differences were observed in all indicators between the FT and TRT groups (*P* > 0.05). Both groups showed significant improvement in time-related outcomes except for fatigue and flexibility (*P* > 0.05). There was significant progress in both groups in aerobic power, muscle power, speed, agility, reaction time, lower body strength, upper body strength, dynamic balance of the right leg, and dynamic balance of the left leg indices from pre to post-tests. The FT group displayed significant improvement in peak power (*P* = 0.006) and mean power (*P* = 0.015) from pre- to post-test.

**Conclusion:**

Both interventions improved most biomotor abilities in elite female taekwondo athletes. It should be noted that since muscle power is vital for taekwondo, therefore, it is recommended to include FT in the taekwondo workout program.

## Introduction

Taekwondo has been one of the Olympic disciplines since the 2000 Olympic Games in Sydney [1]. It is currently held in more than 210 countries worldwide [2]. Taekwondo competitions include three rounds, each for 2 min, separated by 1-min rest between rounds in the Olympics [1–4]. The winner is the fighter who completes two out of three rounds in his/her favor [5]. Athletes may have to fight in 4–5 matches in one day to reach the final match; as such, they encounter excessive physiological fatigue during the competition [3, 4]. Therefore, taekwondo can be described as a high-intensity combat sport, to defeat the opponent by using accurate hand and foot strikes [6]. The sport includes a wide range of high-intensity (1–5 s) aggressive techniques, followed by long periods of low-intensity defensive activity. High to low-intensity activity ratios range from 1:2 to 1:7 [7], indicating the intermittent nature of taekwondo competitions in terms of intensity [8, 9]. Therefore, it seems that taekwondo athletes need to acquire a high anaerobic power, especially in the lower extremities to execute numerous quick and powerful attacks and counterattacks with short recovery periods [10]. On the other hand, high values of maximum oxygen consumption (VO_2_max), heart rate, and blood lactate concentration during taekwondo competitions demonstrate the importance of aerobic metabolism to elite taekwondo athletes [11]. Taekwondo training and competitions also include many high, fast and jump kicks that require superior balance abilities and motor responses of the athletes [12–14]. Therefore, taekwondo athletes need to have extremely good biomotor capabilities, including aerobic and anaerobic fitness, muscle strength, speed, reaction time, agility, lactate production and clearance, body balance, and flexibility [7, 10, 11, 15].

Resistance training (RT), which increases hypertrophy [16], maximum strength [16–19], and hence muscle power, is often an integral part of the athlete's long-term development plans [20]. Therefore, this is an appropriate and pre-requisite method for enhancing muscle power [21]. In this regard, Lesinski et al. (2017) reported that RT increases muscle strength and vertical jump in young athletes, and improves linear running speed, agility, and performance [22]. Improved strength may also affect anaerobic power indicators such as speed and agility [23, 24]. Cin et al. (2021) reported that a six-week of traditional resistance training (TRT) significantly increased some parameters such as speed, agility, maximum strength, and vertical jump in professional volleyball players [24]. While, one study demonstrated that RT has negative effects on cardiovascular indicators such as VO_2_max, lactate threshold, and long-term oxidative enzyme activity [25]. In addition, studies have shown that RT affects speed and agility [19, 26, 27], vertical jump [27, 28], acceleration time [29], anaerobic capacity [30], long jump [27], and change-of-direction speed (COD) [31]. In contrast, a study revealed that RT has not altered the speed and agility of female volleyball athletes [26]. Since elite taekwondo athletes demand more power and speed than maximum strength, functional training has become popular with a different approach to improving power.

Functional training (FT) is a new training approach based on performance, and it has been proposed as a superior alternative to TRT in order to improve biomotor abilities such as strength, speed, power, endurance, and coordination [32, 33]. Furthermore, one of the characteristics of FT is the combination of upper and lower body movements, the use of unstable surfaces, and movement control. Thus, contrary to TRT and strength training, FT avoids focusing on specific muscle adaptations [34, 35]. It has been demonstrated that FT improves aerobic power, anaerobic power, body composition, and muscle strength [36–38]. In this regard, Xiao et al. (2021) found that FT has a significant effect on speed, muscle strength, power, balance, and agility [39]. Besides, Park (2019) revealed that a six-week FT program has significant effects on aerobic and anaerobic power, body composition, as well as strength-related factors such as standing long jump and Sargent's jump in elite taekwondo athletes [40]. Weiss et al. (2010) showed that there is no significant difference between the FT and TRT protocols on some biomotor abilities in young adults [32]. However, so far, the effects of these two resistance training models on performance have not been compared in athletes.

Taekwondo athletes need resistance training to improve their performance. FT and RT can lead to improved performance, but it is crucial to select the most beneficial training model given the limited training time. As mentioned, FT has more neuromuscular facilitation and results in higher power, while RT also has beneficial effects on strength and power, but so far, the literature has not determined whether one of these two training models is more efficient for elite athletes. Hence, this study attempts to answer the question if there is a difference in performance improvement between FT and TRT in elite female taekwondo athletes, and which one is best suited for taekwondo athletes to improve biomotor indicators. We hypothesized that there should be differences in the improvement of biomotor indices between these two training models.

## Methods

### Study design

A semi-experimental trial with a pre-posttest design using two parallel experimental groups was conducted on elite taekwondo female athletes. The main focus of the study was to compare the effects of eight weeks of functional training with traditional resistance training on the biomotor abilities of elite female taekwondo athletes. Participants attended a familiarization session one week before the study and received the necessary information on the objectives, methodology, benefits, and potential risks of the study. “Informed" consent was obtained from all participants. They completed a health and sports history questionnaire and conducted a series of fitness tests. Participants were divided into two groups of functional exercises (*n* = 9) and traditional resistance exercises (*n* = 8) using a simple drawing randomized method.

#### Participants

Elite female taekwondo athletes (1st to 3rd place in national or international competitions over the last 3 to 5 years, participating in Premier League competitions for 5 years) from Kermanshah province were invited to take part in the research. In addition to being elite, the inclusion criteria were more than 18 years old, with no previous serious injuries, and having a history of resistance training. The exclusion criteria were absence for more than three consecutive sessions, physical injury during research, infection by the coronavirus, and refusal to continue research. The sample size corresponded with the statistical population. Twenty eligible participants volunteered to participate in the study, but data for 17 athletes (mean age ± standard deviation: 21.7 ± 3.0 years; mean height ± standard deviation: 167.2 ± 6.1 cm) were eventually analyzed. Three of the participants declined to continue the research (infected by the coronavirus and not willing to continue). A third person who was not in the research team randomly assigned participants into two groups by drawing, including functional training (FT, *n* = 9) and traditional resistance training (TRT, n = 8). All procedures conducted in the study involving human participants were under the Helsinki Statement regarding human research. The Ethics Committees of the Sport Sciences Research Institute of Iran approved the study (Approval Number: IR.SSRI.REC.1401.1664).

## Measurements

### Anthropometric indices

Height and body mass were measured using a standard stadiometer (Seca 206, Germany) and a calibrated digital scale (Seca 769, Germany). Body mass index (BMI) was measured using a body composition instrument (ZEUS 9–9, JAWON, South Korea). Body fat percentage by caliper (SH020, SAEN, South Korea) by taking 7-site skinfolds (chest, abdominal, thigh, triceps, subscapular, suprailiac, and midaxillary) was calculated via the formula [41]:

$$\mathrm{BF}\%=\;495/((1.097 - (0.00046971 \times X) + (0.00000056\times X^{2} - (0.00012828 \times Y))-450.$$

In this formula, X is the sum of 7-site skinfold (mm), and Y is age (year).

### Performance tests

The performance tests were measured in three days. To assess intraclass correlation (ICC), the tests were repeated at 5-day intervals. All assessments were conducted at the Exercise Physiology Laboratory at Razi University. The average temperature of the room was 23ºC (19-24ºC) and the relative humidity of 44% (40–46%). The same conditions were ensured in the pre- and post-training interventions.

The sit and reach test, the Y balance test (YBT), and a Wingate test were assessed on the first day, respectively. The sit and reach test assessed lower back and hamstring flexibility [42]. Participants sit on the floor with their legs stretched out and their soles placed flat against the box. The knees have to be locked and pressed flat on the ground. With the palms facing downwards and the hands on top of each other, participants reached forward and tried to pass their toes. Participants should keep this position for at least 2 s while a tester records the distance. The level of the feet was considered as recording zero so that any measure that does not reach the toes is negative and any reach past the toes is positive. (ICC = 0.97; *P* = 0.001). The Y balance test (YBT) for assessing dynamic balance and core control was performed on the stance plate [43]. Participants stood on one leg while reaching out in three different directions (anterior, posteromedial, and posterolateral) with the other lower extremities. The free foot must be returned to the starting position under control. Participants performed this test twice, and the best trial was recorded (average ICC for each side; ICC = 0.81, *P* = 0.001). The distance read from the test device. To obtain peak power (PP), average power (AP), and fatigue index (FI), a Wingate test on a Monark bike (Model 894E, Monark, Vansbro, Sweden) was performed. After a warm-up, cycling for four min at 60 rpm, the participants were asked to pedal as fast as they could for five seconds, and then by applying a resistance load, they pedaled for 30 s with maximum effort. The workload for the test was equivalent to 0.075 kg per kg body weight. Blood lactate from the forefinger was measured with a lactometer (Cosmos Sirius, Germany). The lactate levels were evaluated at baseline, after warming up, immediately, 5, and 30 min after the Wingate test.

On the second day, after a good warm-up for 15 min, the explosive power, speed, agility, and coordination tests were taken, respectively. The speed test involves running a single maximum sprint over 30 m. The time was recorded by photocell. Start in a stationary position, one foot ahead of the other and behind the start line. Two trials are allowed, and the best time is recorded (ICC = 0.92, *P *= 0.001). The agility T-test was performed to measure the ability of players to change direction in forward, lateral, and backward running. Four cones were set out as illustrated in the T-test instruction [44]. The start and finish line is at cone A, so photocells were located in cone A. Participants start at cone A, sprint to cone B, and touch the base of the cone with their right hand. They then turn left and shuffle sideways to cone C, and touch its base with their left hand. Then, shuffling sideways to the right to cone D and touching the base with the right hand. After that, shuffle back to cone B touching with the left hand, and run back to cone A. The test was performed twice, and the best trial was recorded (ICC = 0.90, *P* = 0.001). To obtain the explosive power, the Sargent test (SJT) was carried out [45]. Participants stand with their side to a wall, reach up with the hand closest to the wall, and have the points of their fingertips marked. They then jump vertically as high as possible using their extremities limbs and touch the wall with one hand at the highest point of the jump. The difference between the standing position and the jump height was used as a record. The best of three trials was recorded (ICC = 0.97, *P* = 0.001). Reaction time was measured by an 8-direction reaction time test (DSI, Danesh Salar Iranian Co, Iran). Initially, to decrease the learning effect, each participant repeated the test 3 times. The participant stands on the mat on the number zero (the mat has 9 houses, 3 rows of 3, and the central house is number zero) while simultaneously looking at the monitor facing her; as soon as the number is displayed on the monitor, the participant must go to the corresponding house. The software of the device randomly selected and displayed 8 numbers. Finally, the record of the participant was displayed on the digital screen of the device in seconds and the scores were recorded by the examiner (ICC = 0.88, *P* = 0.001). The best of two attempts was used as the individual record. In the afternoon, muscle strength of bench press (ICC = 0.91, P = 0.001) and squat (ICC = 0.95, *P* = 0.001) were measured by the one repetition maximum (1RM) using the Berzyski formula [46]. Core muscle endurance (through the 60-s sit-up test), and shoulder girdle endurance (through the 60-s push-up test) was measured and recorded [44].

On the third day, aerobic endurance was measured through the Bruce test. The test was conducted on a treadmill (h/p/cosmos pulsar 3p 5.0, Germany), a speed of 2.7 km·h − 1, and a gradient of 10% for 3 min. Then, workloads (speed and inclination) were increased every 3 min simultaneously until volitional exhaustion, according to the procedures. VO_2_max was measured directly using a portable respiratory gas analyzer device (METAMAX 3B, Germany). The METAMAX 3B was calibrated according to the manufacturer's recommendations before each test [47]. Covered distance, post-heart rate, and VO_2_max were the variables measured in this test. In addition, blood lactate was gathered from the forefinger four min after the Bruce test. Also, 48 h after completing the research protocol, the post-tests were measured in three days according to the pre-test process.

### Training protocol

Both training interventions lasted two months, three sessions per week (24 workouts). Each session lasted 75–90 min, including 10 min of warm-up with dynamic movements, 60–75 min of main exercises, and five min of cool down with stretching movements. In addition, both groups strengthen the core muscles in the same way toward to end of the workout (plank, 30–60 s, 3–4 reps). To determine the exercise loads, one repetition maximum (1RM) of all resistance exercises (TRT group) was calculated based on the instruction and Brzycki formula [46] $\left(1\;RM=\;\frac{\mathrm{weight}}{1.0278\;-\;(0.0278\;\times\;\mathrm{repetition})}\right)$. 1RM was measured at the beginning, the fourth week, and the end of interventions, and the training programs were tailored accordingly. The resistance training program of the TRT group included the Smith squat, barbell chest press, leg extension, lat pull-down, lying leg curl, machine shoulder press, and cable lateral raise. Details on exercise protocol and overload are provided in Table 1. The functional training of the FT group included burpees, barbell squats + standing calf raise, alternate push up on a medicine ball, snatch, clean and jerk, lunge + holding medicine ball, and kettlebell single-leg deadlift. The overload depends on the exercise; for burpee and push-up, the duration with maximum effort increased from 10 to 40 s, in addition, jump height and steppe height increased, and for other exercises, resistance weight increased based on 1RM. The technical training of taekwondo in both groups was carried out in the same manner, three sessions per week. The intensity of the exercise in this section was monitored using a Borg scale (a 20-point scale). The intensity of the exercise on this scale was average to hard (13 to 16).

**Table 1:** Exercise training protocol in the resistance training group

Week	Exercise load (%1RM)	Set (n)	Repetition (n)	Rest (s)
**First**	%50	3	14	60
**Second**	%60	3	12	60
**Third**	%70	4	10	75
**Fourth**	%70	4	10	60
**Fifth**	%60	3	12	60
**Sixth**	%70	4	6	90
**Seventh**	%80	5	6	120
**Eighth**	%80	5	6	120

*1RM*: One-Repetition Maximum

### Statistical analysis

The Statistical Package of Social Sciences (SPSS, IBM, v24) was utilized for data analysis. Data presented in mean ± standard deviation (SD). We determined the normality distribution of the variables using the Shapiro–Wilk test. A repeated measure analysis of variance (ANOVA) with the time (T1 vs. T2) and group (TRT, FT) was performed to analyze the data. Paired T-test was also used to determine the intra-effect of each intervention. An Independent T-test was performed to determine the difference between groups at blood lactate level in the measured times. We calculated the effect size (ES) by the change score divided by the SD of the change score to examine the magnitude of differences while controlling for the influence of the sample size [48], with 0.2 considered as a small ES, 0.5 as a moderate ES and > 0.8 as a large ES. Percentage change was calculated by the formula: $c\mathrm h\mathrm a\mathrm n\mathrm g\mathrm e\;\%=\;\frac{\mathrm{posttest}\;-\;\mathrm{pretest}}{\mathrm{pretest}}\;\times\;100$. The significance level was assumed to be 0.05 for all statistical analyses.

## Results

Table 2 presents the descriptive statistics of anthropometric parameters, pre- and post-interventions. There were no significant differences in demographic indicators such as body mass (t = 0.55, *p* = 0.592), BF% (t = 0.27, *p* = 0.789), BMI (t = 0.38, *p* = 0.705) and FFM (t = 0.62, *p* = 0.543) between the two groups in the pre-test.

**Table 2:** Descriptive statistical values of the anthropometric characteristics of the participants in the pre-and post-test (MEAN ± SD)

		Age (year)	Height (cm)	Weight (kg)	BMI (kg/m^2^)	BF%	FFM
**FT**	Pre	21.1 ± 2.9	167.8 ± 7.3	61.6 ± 7.1	21.8 ± 2.1	21.5 ± 3.1	17.1 ± 1.1
	Post	---	---	61.2 ± 8.2	21.7 ± 2.2	19.2 ± 2.1	17.5 ± 1.4
**TRT**	Pre	22.3 ± 3.1	166.4 ± 4.7	59.8 ± 8.6	21.5 ± 1.5	22.5 ± 2.9	16.7 ± 1.4
	Post	---	---	59.2 ± 7.1	21.3 ± 1.6	19.5 ± 3.3	17.1 ± 1.1

*FT* Functional Training, *TRT* Traditional Resistance Training, *FFM* Fat-Free Mass, *BF%* Body fat percentage, *BMI* Body Mass Index

There were no significant main group (F_1,15_ = 0.16, *p* = 0.695, *ηp*^*2*^:0.01), time (F_1.15_ = 2.29, *p* = 0.150, *ηp*^*2*^:0.13), and interaction effect (F_1.15_ = 0.34, *p* = 0.856, *ηp*^*2*^:0.01) for BMI. We observed a significant main time effect (F_1,15_ = 52.23, *p* = 0.001, *ηp*^*2*^:0.78) but no significant main group (F_1,15_ = 0.07, *p* = 0.769, *ηp*^*2*^:0.01), and interaction effect (F_1.15_ = 0.01, *p* = 0.903, *ηp*^*2*^:0.01) for BF%. In addition, we demonstrated a significant main time effect (F_1,15_ = 15.22, *p* = 0.001, *ηp*^*2*^:0.50) but no significant main group (F_1,15_ = 0.38, *p* = 0.548, *ηp*^*2*^:0.02), and interaction effect (F_1.15_ = 0.06, *p* = 0.812, *ηp*^*2*^:0.01) for FFM. BF% and FFM decreased significantly in both experimental groups (*p* < 0.01).

**Fig. 1:**
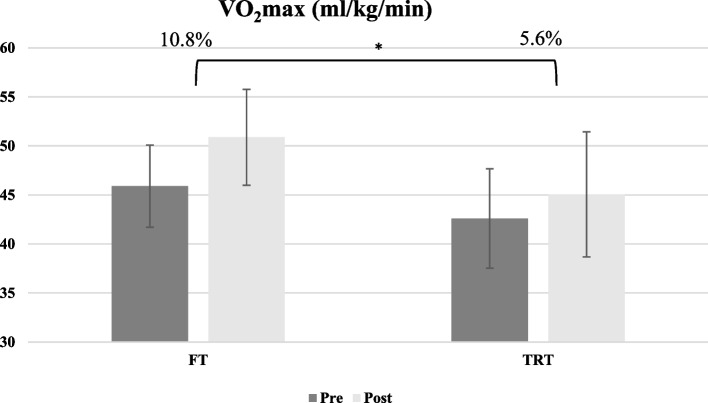
Aerobic power values of participants from pre-test to post-test. *p < 0.05

Overall, both experimental groups demonstrated substantial improvements in all performance indices following interventions. In most indicators, the effect of time was significant, that is, both protocols have been effective in improving performance indicators from pre to post-interventions. As shown in Fig. 1, for aerobic power, there was no significant main group effect (F_1,15_ = 3.46 *p* = 0.083, *ηp*^*2*^:0.18), through a significant time (F_1,15_ = 47.98 *p* = 0.001, *ηp*^*2*^:0.76) and interaction effect (group × time) observed (F_1.15_ = 5.54 *p* = 0.033, *ηp*^*2*^:0.27).

**Fig. 2:**
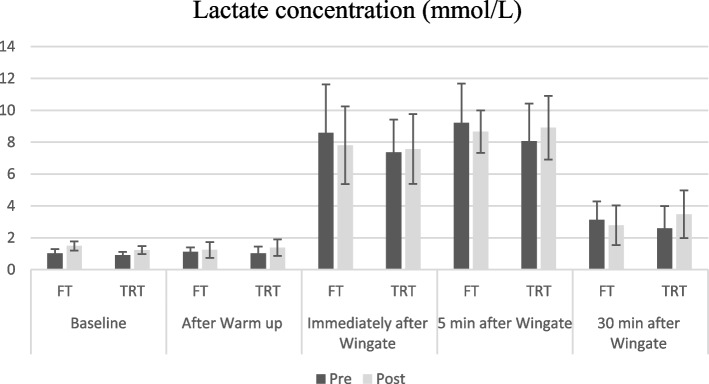
Lactate concentration before and after Wingate test

There was a significant main time effect (F_1,15_ = 8.62, *p* = 0.010, *ηp*^*2*^:0.36), but no main group (F_1.15_ = 1.33 *p* = 0.266, *ηp*^*2*^:0.08), and interaction effect (F_1.15_ = 0.21 *p* = 0.656, *ηp*^*2*^:0.01) for anaerobic peak power (PP) following interventions. For anaerobic mean power (MP), there was a significant main time effect (F_1,15_ = 13.88, *p* = 0.002, *ηp*^*2*^:0.48), but no main group (F_1.15_ = 0.65 *p* = 0.432, *ηp*^*2*^:0.04), and interaction effect (F_1.15_ = 0.29, *p* = 0.598, *ηp*^*2*^:0.29). We observed no significant main group (F_1,15_ = 0.06, *p* = 0.801, *ηp*^*2*^:0.01), time (F_1,15_ = 1.06 *p* = 0.319, *ηp*^*2*^:0.06) and interaction effect (F_1.15_ = 0.91, *p* = 0.356, *ηp*^*2*^:0.06) for fatigue index (FI). In addition, the result of the independent t-test demonstrated that there were no significant differences between the two groups in blood lactate levels at baseline (t = 1.64, *p* = 0.394), after warm-up (t = -0.59, *p* = 0.787), immediately (t = 0.21, *p* = 0.555), 5 (t = -0.313, *p* = 0.204) and 30 min (t = -1.03, *p* = 0.566) following Wingate test (Fig. 2).

The statistical analysis demonstrated a significant main time effect for speed (F_1,15_ = 34.53, *p* = 0.001, *ηp*^*2*^:0.70) and agility (F_1,15_ = 38.19 *p* = 0.001, *ηp*^*2*^:0.72), but no main group for speed (F_1,15_ = 0.06 *p* = 0.815, *ηp*^*2*^:0.01) and agility, and interaction effect (F_1.15_ = 0.47, *p* = 0.506, *ηp*^*2*^:0.03) (Table 3). For agility, there was also a significant main time effect (F_1,15_ = 38.19 *p* = 0.001, *ηp*^*2*^:0.72), but no main (F_1,15_ = 0.01 *p* = 0.915, *ηp*^*2*^:0.01), and interaction effect (F_1.15_ = 0.97, *p* = 0.339, *ηp*^*2*^:0.06). The statistical analysis demonstrated there was a significant main time effect for muscle power (SJT) (F_1,15_ = 42.47, *p* = 0.001, *ηp*^*2*^:0.74), but no main group (F_1,15_ = 1.21 *p* = 0.289, *ηp*^*2*^:0.07), and interaction effect (F_1.15_ = 0.73, *p* = 0.406, *ηp*^*2*^:0.05) (Table 3).

**Table 3:** Performance values of participants from pre to post-test (MEAN ± SD)

Variable	Group	Pre	Post	% change	Cohen’s d	P between group
**Peak Power (w/kg)**	FT	8.79 ± 1.34	9.25 ± 1.24	%5.5	1.24	0.656
	TRT	8.19 ± 1.31	8.53 ± 0.84	%5.1	0.48	
**Average Power (w/kg)**	FT	6.15 ± 0.53	6.47 ± 0.54	%5.3	1.03	0.598
	TRT	5.95 ± 0.81	6.18 ± 0.64	%4.5	0.77	
**Fatigue Index (%)**	FT	57.09 ± 9.16	57.02 ± 7.74	%0.43	-0.01	0.356
	TRT	57.09 ± 6.52	55.18 ± 6.59	%-3	-0.48	
**Sprint (sec)**	FT	5.66 ± 0.31	5.35 ± 0.26	%-5.4	-1.46	0.506
	TRT	5.66 ± 0.31	5.41 ± 0.30	%-4.3	-1.37	
**Agility (sec)**	FT	11.67 ± 0.68	10.94 ± 0.46	%-6.2	-1.52	0.339
	TRT	11.55 ± 0.61	11.01 ± 0.45	%-5.4	-1.55	
**Vertical Jump (cm)**	FT	41.69 ± 5.97	45.05 ± 5.71	%8.3	1.78	0.406
	TRT	38.50 ± 4.15	42.87 ± 4.42	%11.6	1.48	
**1RM bench press (kg)**	FT	35.67 ± 4.81	41.33 ± 5.84	%16	1.80	0.512
	TRT	34.30 ± 5.01	38.95 ± 5.01	%14.1	1.55	
**Reaction time (sec)**	FT	6.70 ± 1.55	5.44 ± 0.94	%-17.7	-1.47	0.127
	TRT	6.25 ± 0.70	5.53 ± 0.56	%-11.2	-1.63	
**Sit-and-reach (cm)**	FT	48.36 ± 5.56	48.86 ± 5.27	%1.1	0.58	0.769
	TRT	46.46 ± 5.46	46.84 ± 4.98	%0.95	0.44	

*FT* Functional Training, *TRT* Traditional Resistance Training

**Fig. 3:**
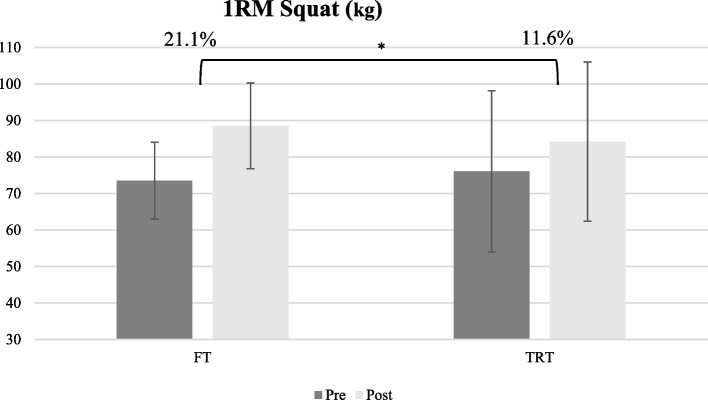
Lower body muscle strength values of participants from pre-test to post-test. *p < 0.05

The statistical analysis for the lower body maximum strength test showed no significant main group (F_1,15_ = 0.12 *p* = 0.915, *ηp*^*2*^:0.01), but a significant main time effect (F_1,15_ = 67.18, *p* = 0.001, *ηp*^*2*^:0.82), and interaction effect (F_1.15_ = 5.90, *p* = 0.028, *ηp*^*2*^:0.28) (Fig. 3). Both groups displayed significant increases in 1RM from pre- to post-test (*p* < 0.01), while the increased value in 1RM in the FT group (21.1%) was greater than the TRT group (11.6%). However, for upper body maximum strength, the result showed there was no significant main group F_1,15_ = 0.60 *p* = 0.449, *ηp*^*2*^:0.04), and interaction effect (F_1.15_ = 0.45, *p* = 0.512, *ηp*^*2*^:0.02), while there was a significant time effect (F_1,15_ = 47.85, *p* = 0.001, *ηp*^*2*^:0.76). The result of the paired t-test showed chest press strength increased in both groups (*p* < 0.05) roughly to the same level.

For reaction time, there was also a significant main time (F_1,15_ = 34.42 *p* = 0.001, ηp^2^:0.70), but no main group (F_1,15_ = 0.14 *p* = 0.712, ηp^2^:0.01) and interaction effect (F_1,15_ = 2.60 *p* = 0.127, ηp^2^:0.15) was observed. The reaction time improved similarly in both groups. For flexibility, sit and reach test, the result showed there was no significant main group (F_1,15_ = 0.57 *p* = 0.461, *ηp*^*2*^:0.04), time(F_1,15_ = 4.37 *p* = 0.054, *ηp*^*2*^:0.22) and interaction effects(F_1,15_ = 0.09 *p* = 0.796, *ηp*^*2*^:0.01) following interventions.

The results of dynamic balance are presented in Table 4. For the dynamic balance of the right leg, a significant main time (F_1,15_ = 223.1 *p* = 0.001, *ηp*^*2*^:0.94), but no main group (F_1,15_ = 0.31 *p* = 0.587, *ηp*^*2*^:0.02) and interaction effect (F_1,15_ = 0.03 *p* = 0.852, *ηp*^*2*^:0.01) was observed. The results were similar for the left leg; there was a significant main time (F_1,15_ = 226.17 *p* = 0.001, *ηp*^*2*^:0.938), but no main group (F_1,15_ = 0.24 *p* = 0.633, *ηp*^*2*^:0.01), and interaction effect (F_1,15_ = 0.08 *p* = 748, *ηp*^*2*^:0.01). Both interventions improved equally dynamic balance in both legs.

**Table 4:** Average dynamic balance of participants from pre to post-test (MEAN ± SD)

Variable	Group	Pre	Post	% change	Cohen’s d	P between group
**Average Balance Anterior Right (cm)**	FT	92.94 ± 5.78	105.62 ± 5.45	%13.8	3.62	0.125
	TRT	89.66 ± 3.37	105.61 ± 6.49	%17.8	3.33	
**Average Balance Posteromedial Right (cm)**	FT	98.07 ± 7.59	114.23 ± 6.74	%16.8	2.87	0.264
	TRT	98.45 ± 6.30	110.38 ± 11.84	%12.1	1.29	
**Average Balance Posterolateral Right (cm)**	FT	101.04 ± 6.39	118.25 ± 5.64	%17.2	3.94	0.395
	TRT	104.94 ± 6.16	120.46 ± 6.75	%14.9	4.43	
**Composite Balance Right (cm)**	FT	109.70 ± 10.49	127.02 ± 11.55	%15.9	4.91	0.852
	TRT	113.13 ± 10.85	130.02 ± 15.44	%14.7	2.92	
**Average Balance Anterior Left (cm)**	FT	90.41 ± 4.96	102.67 ± 5.04	%13.62	4.25	0.904
	TRT	90.20 ± 4.78	102.22 ± 7.88	%13.26	2.41	
**Average Balance Posteromedial Left (cm)**	FT	98.02 ± 6.19	112.70 ± 5.71	%15.28	2.09	0.285
	TRT	98.36 ± 7.64	109.74 ± 10.22	%11.52	2.37	
**Average Balance Posterolateral Left (cm)**	FT	98.37 ± 5.05	113.99 ± 5.17	%15.95	5.28	0.153
	TRT	97.25 ± 8.69	116.50 ± 5.93	%20.732	2.94	
**Composite Balance Left (cm)**	FT	107.78 ± 10.74	123.75 ± 11.79	%14.9	3.90	0.748
	TRT	110.39 ± 12.01	126.96 ± 15.16	%14.9	3.43	

## Discussion

The purpose of this study was to compare eight weeks of FT and TRT on the biomotor capacities of elite female taekwondo athletes. It is important to note that most of the factors improved from pre-test to post-test in both training protocols, except fatigue index and flexibility. In addition, the peak and average anaerobic power significantly increased after 8 weeks in the FT group compared to the TRT group. Therefore, the research assumption applies only to anaerobic power, and in other biomotor capacities, both training models were equally effective.

Both interventions resulted in improvements in aerobic power. Although there was no statistical difference between groups, there was a greater improvement in the FT group compared to the TRT group (10.8% vs. 5.6%). However, it should be noted that at the elite level, these small differences determine success [49]. Previous studies have confirmed these results and have shown that 6 and 4 weeks of FT and RT training combined with regular taekwondo training enhances VO_2_max, exercise duration, and combat performance [40, 50]. In contrast, no improvement in VO_2_max was reported in a study following strength training in trained female cyclists [51]. Methodological differences may explain the inconsistency. In the last study, two sessions per week were conducted, while in the research that reported an improvement, three sessions per week were performed. Also, the subjects were cyclists who had a high initial aerobic fitness level. Therefore, the fitness level of subjects and the type of sports may affect the results of the research.

Results from our study showed that both FT and TRT training interventions improved peak (5.5% vs. 5.1%) and average (5.3% vs. 4.5%) anaerobic power, but only in the FT group, this improvement was significant; although there was no statistical difference between groups. Also, there were no significant intra- and inter-group differences. The studies using FT [40, 52] and RT [53, 54], yielded results consistent with our study. Park (2019) showed that 6 weeks of FT with a frequency of 3 sessions per week leads to improved peak and average anaerobic power of elite taekwondo athletes [40]. Besides, Teng et al. (2008) revealed that 2 sessions per week of RT for 12 weeks improve the peak (10%) and average anaerobic power (9%) of young taekwondo players [54]. None of these studies examined the fatigue index. Since FTs are multi-joint movements and involve several muscle groups, they create more neuromuscular coordination and are probably involved in improving anaerobic power. The fatigue index improved to an equal extent in both groups, which was favorable for the TRT group (3% vs. 0.48%). The greatest improvement in minimum power in the TRT (6.8% in the FT and 10% in the TRT) was the cause of the non-significant difference between the two groups. According to the fatigue index formula, this factor has caused a greater reduction of the numerator in TRT and consequently, a further improvement of the fatigue index. Moreover, no significant differences were observed between the two groups in the times at which lactate was measured. Although, lactate drop values in the post-test were higher in the FT group. Involving more muscles and more blood flow in the FT workout is probably the reason for faster removal of lactate.

Both interventions led to similar improvements in reaction time (17.7% vs. 11.2%), speed (5.4% vs. 4.3%), and agility (6.2% vs. 5.4%) which is in favour of the FT group. Improvements in speed and agility have been reported in previous studies using FT [52, 55, 56] and TRT [24, 29, 55] exercises. Cin et al. (2021) reported that six weeks of TRT greatly improved the speed and agility parameters of professional volleyball players [24]. There has been less research about reaction time. Redondo et al. (2014) studied the effect of 12 weeks of strength training, and twice-weekly sessions on elite fencers and noticed no change in reaction time [57]. Given the great importance of reaction time in the success of fencers, fencers may be said to have good initial reaction time values, and improving it requires years of practice. Improving the neuromuscular function and increasing the recruitment of fast-twitch fibers can be the factor in improving the reaction speed. The main factor in improving speed and agility may be related to neuromuscular adaptations caused by exercise. Neural adaptations, such as increased synchronicity of muscle fiber recruitment, lead to more power generation and improved speed and agility. It appears that the greatest improvement in the FT group may be attributed to the greater neuromuscular coordination of FT. In contrast, some studies have shown that TR has no effect on speed and agility [26, 27, 58] and FT has no effect on speed [58]. The fitness level of subjects and the type of sports may be the reason for the discrepancy in these studies.

A similar improvement in lower body and upper body maximal strength as well as explosive power was observed after training interventions. This finding was not unexpected, because most studies of FT [32, 40, 55, 56] and RT [24, 26, 32, 55] have reported an improvement in maximal strength. Park (2019) reported an improvement in lower body strength and explosive power in elite taekwondo athletes after 6 weeks of FT with a frequency of 3 sessions per week [40]. On the contrary, some investigations [53, 58] demonstrate no remarkable effects on explosive power by FT or RT. Brown et al. (2007) showed that 6 weeks of RT, twice a week, despite the significant improvement in lower body strength, did not affect the explosive power of female dancers [53]. The duration, intensity, frequency, and volume of training are the variables that affect the strength and explosive power; these indicators have been different between the mentioned researches. Although the increase in upper body maximal strength was the same in both groups (16% in FT and 14.1% in TRT), the increase in lower body maximal strength was greater in FT (21.1% in FT and 11.6% in TRT). Interestingly, the improvement in explosive power in TRT was higher, despite greater improvement in lower body strength in FT (8.3% in FT and 11.6% in TRT). Considering the composition of muscle fibers and their elastic properties, it is possible that the rate of force development in TRT was slightly higher than in FT. Overall, it is concluded that the FT and TRT had the same effect on the maximum upper and lower body strength and explosive power.

Both interventions improved the dynamic balance performance of both legs. Improvements in the balance ability of elite taekwondo athletes have been reported after 6 weeks of FT [40]. Such improvement in dynamic balance performance may be attributed to the improved postural control strategies and knee muscular performance after FT [59]. Yoo et al. (2018) also revealed that 3 sessions per week of lower body RT for 6 weeks significantly enhanced the balance of pomsae taekwondo athletes [60]. This discrepancy may return to the type of training program. Since muscular strength is a key factor in enhancing balance, it is likely that both interventions, by increasing the strength, led to an increase in the right leg (15.9% in FT, 14.7% in TRT) and the left leg balance (14.9% in FT; 14.9% in TRT). Overall, the dynamic balance also improves by improving the muscle strength observed during the two interventions.

As expected, training interventions had no remarkable effect on flexibility (1.1% in FT and 0.95% in TRT). Naturally, resistance training is associated with muscle contraction, and even muscle hypertrophy is associated with a decrease in joint range of motion. Song et al. (2014) reported no significant change in flexibility after 16 weeks of FT on elite baseball players [61]. In addition, Christou et al. (2006) reported no significant change in flexibility values after 16 weeks of RT, twice a week in young soccer players [29]. It is worth noting that the level of flexibility of taekwondo players is initially high and stretching exercises are part of the taekwondo workout program. In contrast, some FT [32, 52, 56] and TRT [27, 50] interventions have reported improved flexibility. Mathunjwa et al. (2020) determined that adding 3 sessions per week of RT for 4 weeks to taekwondo training significantly improves the flexibility of taekwondo players [50]. Since stretching is part of the taekwondo workout, maybe its enhancement is related to the taekwondo workout itself.

We acknowledge that there were some limitations in the research we conducted. Firstly, during the Corona pandemic, it was a challenge to acquire more elite taekwondo athletes (the national team), and only the elite athletes from one province were willing to collaborate with us. Secondly, Even though all physical fitness indicators were measured, the validity of the research would be enhanced if the specific aerobic and anaerobic tests for Taekwondo were taken. Finally, this research was aimed to investigate the changes in performance, but if possible, researchers are advised to use ultrasound technology to accurately determine muscle size and also blood sampling to investigate some cytokines related to hypertrophy and muscle strength.

## Conclusion and implication

The findings of this study showed that after 8 weeks of FT and TRT, improvements occurs in most of the biomotor capabilities such as speed, strength, reaction time, agility, aerobic and anaerobic fitness, except fatigue index and flexibility. However, considering the superiority of FT in the improvement of peak and average anaerobic power and the higher percentage of improvement in most indicators, taekwondo athletes and coaches are encouraged to use the FT to improve physical performance.

## Data Availability

Data would be available from the corresponding author on reasonable request.

## Abbreviation

FT: Functional training
TRT: Traditional resistance training
VO_2_max: Maximum oxygen consumption
RT: Resistance training
YBT: Y balance test
ICC: Intraclass correlation
FI: Fatigue index
PP: Peak power
AP: Average power
SJT: Sargent test
1RM: One repetition maximum
ES: Effect sizes
